# Common and unique testosterone and 17 beta-estradiol degradation mechanisms in *Comamonas testosteroni* JLU460ET by transcriptome analysis

**DOI:** 10.3389/fmicb.2023.1238855

**Published:** 2023-10-26

**Authors:** Ze Wang, Mingming Chen, Na Liu, Yongkang Zhao, Jintao Ru, Chuanyu Qin, Tingdi Zhang

**Affiliations:** ^1^Key Laboratory of Groundwater Resources and Environment (Jilin University), Ministry of Education, Jilin Provincial Key Laboratory of Water Resources and Environment, College of New Energy and Environment, Jilin University, Changchun, Jilin, China; ^2^Department of Ecology, College of Life Science and Technology, Jinan University, Guangzhou, Guangdong, China

**Keywords:** *C. testosteroni* JLU460ET, testosterone, 17 beta-estradiol, transcriptome, degradation

## Abstract

Strain *C. testosteroni* JLU460ET was isolated for testosterone and 17 beta-estradiol degradation by our group. In this study, strain *C. testosteroni* JLU460ET was induced by testosterone and 17 beta-estradiol and then subjected to transcriptome analysis. There were 2,047 upregulated genes after 3 h of testosterone induction, 2,040 upregulated genes after 13 h of testosterone induction, 2,078 upregulated genes after 3 h of 17 beta-estradiol induction, and 2,095 upregulated genes after 13 h of 17 beta-estradiol induction. Significantly upregulated genes were mainly involved in steroid and aromatic compound degradation. A 100 kb steroid-degrading gene cluster was found by transcriptome analysis, which included 92 annotated genes and 58 novel genes. Among them, MucB/RseB and Fiu are secretory proteins for sensing substrates in the environment. MFS-1 and TonB are transporters of testosterone and 17 beta-estradiol. Ring-cleavage enzymes and beta-oxidation enzymes are important for degradation. The genes upregulated by both substrates were almost the same, but the degree of induction by testosterone was higher than that by 17 beta-estradiol. Nine upregulated genes were selected for verification by quantitative real-time polymerase chain reaction (qRT–PCR). The qRT-PCR results were consistent with the transcriptome sequencing results. In this study, the common and unique metabolic mechanisms of testosterone and 17 beta-estradiol were compared by transcriptome analysis in *C. testosteroni* JLU460ET for the first time.

## Introduction

Steroid hormones, consisting of progestogens, glucocorticoids, mineralocorticoids, androgens, and estrogens, are powerful signaling molecules that regulate a host of organismal functions. Steroid hormones are widely used as medicines for humans and animals, especially in the breeding industry. Consequently, steroid hormone pollution in the environment has become severe and has attracted public attention due to its endocrine-disrupting effects and carcinogenicity (Combalbert and Hernandez-Raquet, 2010; Whitman, 2017; Liu et al., 2020). Among all endocrine-disrupting compounds (EDCs), estrogens have the highest estrogenic activity, and the most recent predicted no-effect concentrations (PNECs) are 0.1 ng/L for 17-alpha ethinylestradiol, 2 ng/L for 17 beta-estradiol, 6 ng/L for estrone, and 60 ng/L for estriol (Caldwell et al., 2012).

Estrogen pollution occurs mainly from wastewater treatment plants (WWTPs) and concentrated animal feeding operations (CAFOs). In slurries from dairy farms, values of approximately 600–1,600 μg estrogen/kg total solid have been reported. In total, 800–1,300 μg of estrogen/kg dry matter manure was found in pregnant cows. Chicken manure contains up to 533 μg estrogen/kg dry matter (Shore and Shemesh, 2003). Concentrations of estrogens in wastewater treatment plants vary from several ng/L to hundreds ng/L. In the natural environment, concentrations of estrogens in surface water were generally below 10 ng/L (Shore and Shemesh, 2003; Combalbert and Hernandez-Raquet, 2010). Estrogens in the environment can interact with physiological systems and cause alterations in development, growth, and reproduction of living organisms, such as fish, mollusks, barnacle, and even plants. For example, the effect on fish ranges from subtle changes in the physiology and sexual behavior to permanently altered sexual differentiation and impairment of fertility. Estrogen contamination can also increase the incidence of cancer in hormonally sensitive tissues in women, precocious puberty in children, and a decreasing reproductive fitness in men (Daston et al., 1997; Juberg, 2000; Partsch and Sippell, 2001). It is necessary to degrade estrogens in severely polluted environment.

Biodegradation using microorganisms is considered one of the most efficient strategies to remove estrogen. Many strains including *Gordonia*, *Sphingomonas*, *Novosphingobium*, *Rhodococcus*, *Acinetobacter*, *Pseudomonas*, *Comamonas*, and *Nocardioides* have been isolated from soil, rivers, oceans, and other environments to degrade estrogen (Yu et al., 2013; Liu et al., 2020, 2021a). Among them, *Comamonas testosteroni* strain JLU460ET isolated by our group could degrade both 17beta-estradiol and testosterone. We found that testosterone is much easier to degrade than 17beta-estradiol in *Comamonas testosteroni* strain JLU460ET. In total, 272 mg/L testosterone could be degraded in 9 h by strain JLU460ET, but 5 mg/L 17beta-estradiol needed approximately 5 days for complete degradation (Liu et al., 2021a).

Both testosterone and 17 beta-estradiol are steroid hormones. They have similar chemical structures with four rings. In bacteria, testosterone can only be degraded using androstenedione as an intermediate followed by B-ring cleavage. Testosterone degradation strains include *Rhodococcus jostii* RHA1, *Mycobacterium tuberculosis* H37Rv, *C. testosteroni* TA441, *C. testosteroni* ATCC11996, and *C. testosteroni* JLU460ET (Horinouchi et al., 2010, 2012; Weiss et al., 2013; Liu et al., 2021a). Among them, the testosterone degradation pathway in *C. testosteroni* strains has been extensively investigated. The complete testosterone degradation pathway has also been well clarified (KEGG map0984). Metabolic intermediates, degradation genes, and regulatory genes have also been studied intensively (Horinouchi et al., 2010, 2012; Ibero et al., 2019). The 17 beta-estradiol degradation pathway is more complex than that of testosterone. At least 4 pathways of 17 beta-estradiol degradation have been reported in bacteria (Liu et al., 2020, 2021b). Some metabolic intermediates and enzymes have been identified, but the whole metabolic pathway and the regulatory genes involved remain unclear (Chen et al., 2017; Wang et al., 2018; Wu et al., 2019; Tian et al., 2020). Most importantly, 17 beta-estradiol is much more resistant to biodegradation than testosterone.

To improve the estrogen biodegradation rate, sometimes genetic engineering is necessary to operate. The testosterone degradation rate is fast, but 17 beta-estradiol degradation rate is slow; the testosterone degradation pathway is very clear, while that of 17 beta-estradiol is not. According to the chemical structures and degradation rates of 17 beta-estradiol and testosterone, *C. testosteroni* strain JLU460ET probably has common genes for their degradation. Identifying the common and unique genes in testosterone and 17 beta-estradiol degradation may help us improve 17 beta-estradiol degradation speed by genetic engineering. To identify the common and unique mechanisms of testosterone and 17 beta-estradiol degradation in *C. testosteroni* JLU460ET, whole-transcriptome analysis was conducted in this article.

## Materials and methods

### Chemicals, enzymes, and kits

Seventeen beta-estradiol (> 98% purity) and testosterone (> 99% purity) were purchased from J&K Scientific Co. (Beijing, China). LB broth and other chemicals were obtained from Sangon Biotech (Shanghai, China). RNAprotect bacteria reagent was from QIAGEN, Germany. PrimeScript Reverse Transcriptase Kit, PrimeScript™ RT Reagent Kit, and TB Green™ Premix Ex Taq™ were from TaKaRa, China. NEBNext Ultra Directional RNA Library Prep Kit was from NEB, China. DNA extraction kit, RNase, and RNase-free DNase I were from Tiangen, China.

### Bacterial culture, growth conditions, and RNA isolation

*C. testosteroni* JLU460ET was isolated from animal waste for estrogen degradation, and it could degrade testosterone and 17 beta-estradiol best at 30°C with pH = 7 (Liu et al., 2021a). In this article, *C. testosteroni* JLU460ET was cultured in LB broth at 30°C. After 10 h of incubation (OD_600_ is approximately 0.8–1.0), testosterone (300 mg/L) and 17 beta-estradiol (10 mg/L) were then added to the fresh culture for induction. After testosterone or 17 beta-estradiol induction, samples were transferred into RNAprotect bacteria reagent (QIAGEN, Germany) for RNA isolation. Altogether, there were six groups containing 18 samples, and each group had three replicates. Group 1 included three samples without induction after 3 h of incubation (named “G1_3_1,” “G1_3_2,” and “G1_3_3”), Group 2 included three samples with testosterone after 3 h of induction (named “G1_T_3_1,” “G1_T_3_2,” and “G1_T_3_3”), Group 3 included three samples with 17 beta-estradiol after 3 h of induction (named “G1_E2_3_1,” “G1_E2_3_2,” and “G1_E2_3_3”), Group 4 included three samples without induction after 13 h of incubation (named “G1_13_1,” “G1_13_2,” and “G1_13_3”), Group 5 included three samples with testosterone after 13 h of induction (named “G1_T_13_1,” “G1_T_13_2,” and “G1_T_13_3”), and Group 6 included three samples with 17 beta-estradiol after 13 h of induction (named “G1_E2_13_1,” “G1_E2_13_2,” and “G1_E2_13_3”). Among all 18 samples, 6 samples of group “G1_3” and group “G1_13” were negative controls at different incubation time points. All treated samples were centrifuged at 4°C for 10 min at 5000 × g. RNA extraction reagent was used for the extraction of total RNA, according to the manufacturer’s guidelines (Tiangen, China). Detailed information on groups, total RNA extraction, and test results is shown in Supplementary Document S1. For the removal of genomic DNA, the RNA samples were treated with RNase-free DNase I (Tiangen, China). RNA degradation and contamination were monitored on 1% agarose gels. RNA integrity was assessed using the RNA Nano 6,000 Assay Kit of the Bioanalyzer 2,100 system (Agilent Technologies, CA, United States).

### Library preparation for transcriptome sequencing

The cDNA libraries for RNA sequencing were constructed by the chain-specific library using the NEBNext Ultra Directional RNA Library Prep Kit (Illumina), according to the manufacturer’s instructions (NEB #E7420S) (Parkhomchuk et al., 2009). Paired-end sequencing was performed on a NovaSeq 6,000 sequencing platform.

### Clustering and sequencing

The clustering of the index-coded samples was performed on a cBot Cluster Generation System using TruSeq PE Cluster Kit v3-cBot-HS (Illumina), according to the manufacturer’s instructions. After cluster generation, the library preparations were sequenced on an Illumina NovaSeq platform, and 150 bp paired-end reads were generated (McLachlan et al., 2008).

### Quality control

Raw reads in fastq format were first processed by in-house Perl scripts. In this step, clean reads were obtained by removing reads containing adapters, reads containing N bases, and low-quality reads from raw data. At the same time, the Q20, Q30, and GC contents in the clean data were calculated. All downstream analyses were based on clean data with high quality (Garber et al., 2011).

### Read mapping to the reference genome

The reference genome and gene model annotation files of strain *C. testosteroni* JLU460ET were downloaded from the NCBI database (genome accession number is CP067086.1). Both the reference genome and aligned clean reads to the reference genome were analyzed using Bowtie2-2.2.3 (Langmead and Salzberg, 2012).

### Novel gene and gene structure analysis

Rockhopper was used to identify novel genes, operons, transcription start sites (TSSs), transcription terminal sites (TTSs), and cis-natural antisense transcripts (McClure et al., 2013). It can be used for efficient and accurate analysis of bacterial RNA-seq data and can aid in the elucidation of bacterial transcriptomes. Then, we extracted the upstream 700 bp sequence of the transcription start site for predicting the promoter using a time delay neural network (TDNN) (Reese, 2001; Suzek et al., 2001). According to the transcription and translation start/terminal site information, we extracted 5’ UTR (3’ UTR) sequences. Then, RBSfinder and TransTermH were used to predict the Shine–Dalgarno (SD) sequence and terminator sequence, respectively (Kingsford et al., 2007).

### Analysis of sRNA

Rockhopper was used to identify new intergenic region transcripts, Blastx was run with the NR library to annotate the newly predicted transgenic regions, and the unmarked transcripts were used as candidate non-coding sRNAs. RNA fold and IntaRNA were used to predict secondary structure and target genes, respectively (Busch et al., 2008; Nawrocki et al., 2015).

### Quantification of gene expression

HTSeq v0.6.1 was used to count the read numbers mapped to each gene. Then, the fragments per kilobase of transcript sequence per million base pairs (FPKM) of each gene was calculated based on the length of each gene and read counts mapped to each gene.

### Differential expression analysis

Differential expression analysis during testosterone and 17 beta-estradiol induction at different time points was performed using the DESeq R package (1.18.0). DESeq provides statistical routines for determining differential expression in digital gene expression data using a model based on the negative binomial distribution. The resulting *p*-values were adjusted using Benjamini and Hochberg’s approach for controlling the false discovery rate. Genes with an adjusted *p*-value of <0.05 found by DESeq were assigned as differentially expressed (Anders and Huber, 2010; Trapnell et al., 2012).

### KEGG enrichment analysis of upregulated expressed genes

Kyoto Encyclopedia of Genes and Genomes (KEGG) is a database resource for understanding high-level functions and utilities of biological systems. KOBAS software was used to test the statistical enrichment of differentially expressed genes in KEGG pathways. Map00984 in KEGG is the pathway for steroid degradation, according to cholesterol and testosterone degradation; map01220 in KEGG is the pathway for the degradation of aromatic compounds. Therefore, mainly the upregulated genes found in KEGG map00984 and map 01220 were analyzed in this study. KEGG pathways related to ABC transporters, the two-component system, fatty acid degradation, and bacterial chemotaxis were also analyzed.

### Quantitative real-time PCR analysis of testosterone and 17beta-estradiol induction genes

For confirmation of the RNA-seq results, nine upregulated genes were selected for validation by qRT–PCR. The criteria for selection of these genes are located in the 106,743-bp steroid degradation gene cluster, and they are significantly upregulated. The selected gene numbers are GM003925 to GM003933. 16S rRNA, reported to be one of the most stable genes, was chosen as a reference gene. The primers for the selected genes were designed, as shown in Supplementary Table S2. Strain JLU460ET was cultured in LB broth at 30°C. After 10 h of incubation, testosterone and 17 beta-estradiol were then added to the fresh culture for induction. After testosterone or 17 beta-estradiol induction, samples at different time points were transferred into RNAprotect bacteria reagent (QIAGEN, Germany) for RNA isolation. The yield of RNA was estimated using a Nanodrop UV spectrometer (Thermo Fisher Scientific, Waltham, MA, United States). Total RNA was treated with the PrimeScript™ RT Reagent Kit with gDNA Eraser (Perfect Real Time) (TaKaRa, Cat. RR047A) for elimination of genomic DNA and reverse-transcribed following the manufacturer’s instruction. The synthesized cDNA was used as a template for qRT–PCR. TB Green™ Premix Ex Taq™ (TaKaRa, Cat. RR820A) was used for qRT–PCR, following the manufacturer’s guidelines. qRT-PCR was performed using the Stratagene Mx3000P instrument. Each reaction was carried out in triplicate with a total reaction mix of 20 μL final volume consisting of 1 μl of 50 ng/μl cDNA, 10 μL of TB GreenTM Premix Ex TaqTM II (Tli RNaseH Plus) (2X), 0.5 μL PCR forward primer (10 μM), 0.5 μL PCR reverse primer (10 μM), 0.4 μL ROX Reference Dye II (50X) *2, and 7.6 μL sterile purified water. qPCR amplification conditions were as follows: The reactions were incubated at 95°C for 1 min, followed by 40 cycles of 95°C for 10 s, 60°C for 30 s, and 72°C for 30 s, with a final melting curve analysis. The sample without reverse transcriptase was used as a negative control. The relative level of expression of the selected genes was normalized to the expression of the reference gene 16S rRNA and computed according to the 2^-△△Ct^ method (Livak and Schmittgen, 2001). At least three independent experiments were conducted. Each experiment had three parallel samples, and the average values were calculated with standard errors.

## Results

### *De novo* assembly of the *Comamonas testosteroni* JLU460ET transcriptome

*C. testosteroni* JLU460ET is a newly reported testosterone- and 17 beta-estradiol-degrading bacterium (Liu et al., 2021a). To investigate the highly expressed genes involved in testosterone and 17 beta-estradiol degradation, we performed whole-transcriptome analysis by using RNA-Seq. All data were submitted to NCBI GEO (accession number GSE184154). Detailed sequencing information and quality control are shown in Table 1; Supplementary Table S3. All sequenced transcripts were used to map annotated genes in the genome sequence of *C. testosteroni* JLU460ET. The percentage of reads mapped to genome regions was approximately 99%, as shown in Table 1.

**Table 1:** Total reads of strain *C. testosteroni* JLU460ET (control and treated) generated after paired end sequencing by using NovaSeq 6,000 platform.

Sample names	Raw reads	Clean reads	Clean bases	Filter rate (%)	Error rate (%)	Q20 (%)	Q30 (%)	GC content (%)	Percent of reads mapped to genome regions (%)
G1_3_1	8,230,602	8,199,752	1.2G	99.63	0.02	98.53	95.47	61.29	99.63
G1_3_2	7,812,094	7,768,666	1.2G	99.44	0.02	98.7	95.98	61.38	99.52
G1_3_3	7,704,174	7,673,262	1.2G	99.6	0.02	98.68	95.89	61.47	99.63
G1_T_3_1	7,552,078	7,508,410	1.1G	99.42	0.02	98.72	96.06	61.81	99.57
G1_T_3_2	7,924,582	7,879,910	1.2G	99.44	0.02	98.56	95.66	61.78	99.58
G1_T_3_3	7,691,636	7,660,190	1.1G	99.59	0.02	98.66	95.92	61.75	99.59
G1_E2_3_1	7,834,010	7,684,738	1.2G	98.09	0.02	98.78	96.31	62.3	99.55
G1_E2_3_2	7,634,556	7,579,498	1.1G	99.28	0.02	98.67	95.97	63.84	99.71
G1_E2_3_3	9,138,000	9,092,676	1.4G	99.5	0.02	98.66	95.96	62.35	99.55
G1_13_1	8,427,262	8,301,680	1.2G	98.51	0.02	98.68	95.99	61.93	99.52
G1_13_2	7,858,654	7,760,232	1.2G	98.75	0.02	98.61	95.84	60.32	96.46
G1_13_3	7,914,712	7,731,364	1.2G	97.68	0.02	98.74	96.15	60.56	99.38
G1_T_13_1	7,620,894	7,590,536	1.1G	99.6	0.02	98.7	96	64.13	99.59
G1_T_13_2	8,469,770	8,412,362	1.3G	99.32	0.02	98.66	95.96	64.09	99.73
G1_T_13_3	7,584,670	7,558,642	1.1G	99.66	0.02	98.65	95.95	64.02	99.66
G1_E2_13_1	7,892,608	7,833,672	1.2G	99.25	0.02	98.67	95.92	64.11	99.75
G1_E2_13_2	7,730,272	7,690,808	1.2G	99.49	0.02	98.59	95.84	64.5	99.75
G1_E2_13_3	7,804,306	7,754,754	1.2G	99.37	0.02	98.67	96.08	65.3	99.52

According to the assembly results, 6,152 genes were found in *C. testosteroni* JLU460ET transcripts, including 5,016 annotated genes, 920 novel genes, and 216 sRNAs. The gene structures predicted in *C. testosteroni* JLU460ET include a 757 SD sequence, 2,544 3’UTR, 2213 5’UTR, 430 terminators, 4,922 operons, and 472 sRNAs (Table 2). The results of gene function classification (GO) analysis showed that the genes found in *C. testosteroni* JLU460ET transcripts were mainly involved in metabolic processes and catalytic activity.

**Table 2:** Gene structures predicted in *C. testosteroni* JLU460ET.

	SD sequence	3’UTR	5’UTR	Terminators	Operons	TSS_TTS	sRNAs
Numbers	757	2,544	2,213	430	4,922	4,922	472

### A 100 kb steroid degradation gene cluster was identified by analyzing the differentially expressed genes under testosterone and 17 beta-estradiol induction

Both testosterone and 17 beta-estradiol are steroids. Seventeen beta-estradiol is synthesized from testosterone in vertebrates. They have similar chemical structures. Some of the testosterone and 17 beta-estradiol degradation pathways in bacteria are the same, but some pathways are different. Normally, genes involved in degradation are upregulated after induction with substrate. To determine the degradation pathways of testosterone and 17 beta-estradiol in *C. testosteroni* JLU460ET, induction of testosterone and 17 beta-estradiol was applied. Induction time points of 3 h or 13 h were considered in this study.

**Figure 1:**
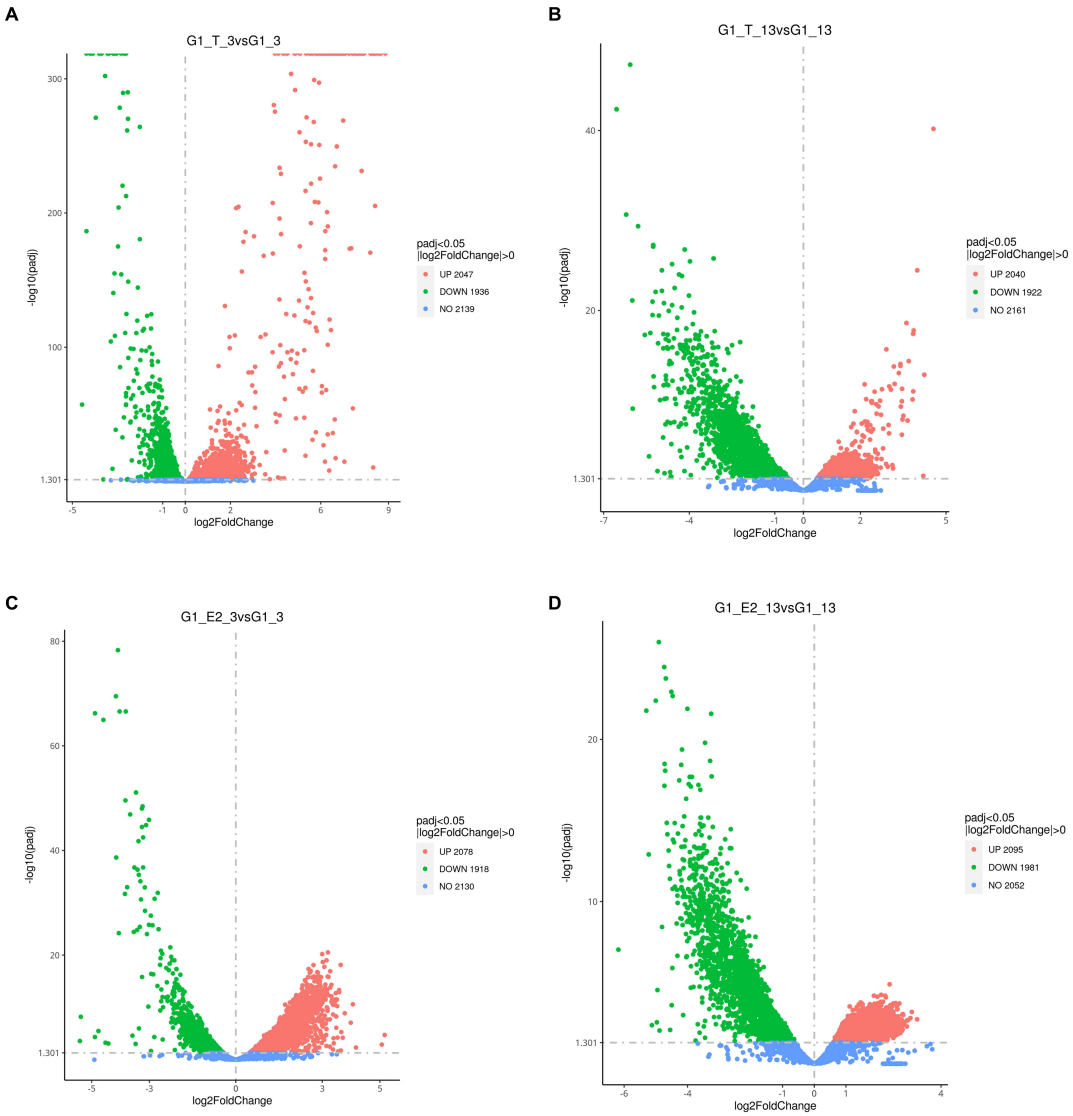
Volcano plot showing the global gene expression profiles (RNA-Seq) of *C. testosteroni* JLU460ET grown under different conditions. **(A)** 3-h induction with testosterone; **(B)** 13-h induction with testosterone; **(C)** 3-h induction with 17 beta-estradiol; **(D)** 13-h induction with 17 beta-estradiol. Green indicates upregulated genes; orange indicates downregulated genes; blue indicates no significant change.

All significant differentially expressed transcripts in *C. testosteroni* JLU460ET induction with testosterone or 17 beta-estradiol are shown in Table 3. The results showed that the number of differentially expressed transcripts was similar among the four compared groups: 2,047 upregulated genes present in 3-h induced samples with testosterone; 2,020 upregulated genes present in 13-h induced samples with testosterone; 2,078 upregulated genes present in 3-h induced samples with 17 beta-estradiol; and 2,095 upregulated genes present in 13-h induced samples with testosterone 17 beta-estradiol. The volcano plot shows that the upregulated log2-fold change value after 3 h of testosterone or 17 beta-estradiol induction was higher than that after 13 h of induction, and the upregulated log2-fold change value induced by testosterone was higher than that induced by 17 beta-estradiol (Figure 1). Altogether, genes induced with testosterone after 3 h of induction had the highest upregulated log2-fold change value among all tested groups. Seventeen beta-estradiol could induce gene expression at the transcriptional level, but the upregulated log2-fold change value was not as high as that induced by testosterone. Detailed analysis showed that 886 annotated genes, 508 novel genes, and 74 sRNAs were induced by testosterone and 17 beta-estradiol at both induction time points. These genes are commonly induced by testosterone and 17 beta-estradiol.

**Table 3:** Significant differentially expressed transcripts in *C. testosteroni* JLU460ET induction with testosterone or 17 beta-estradiol.

Compared groups	Up-regulated transcripts	Down-regulated transcripts	All regulated transcripts	No significance changed transcripts	Total expressed transcripts
G1_T_3 vs. G1_3	2047	1936	3,983	2,139	6,122
G1_T_13 vs. G1_13	2040	1922	3,962	2,161	6,123
G1_E2_3 vs. G1_3	2078	1918	3,996	2,130	6,126
G1_E2_13 vs. G1_13	2095	1981	4,076	2052	6,128

**Figure 2:**
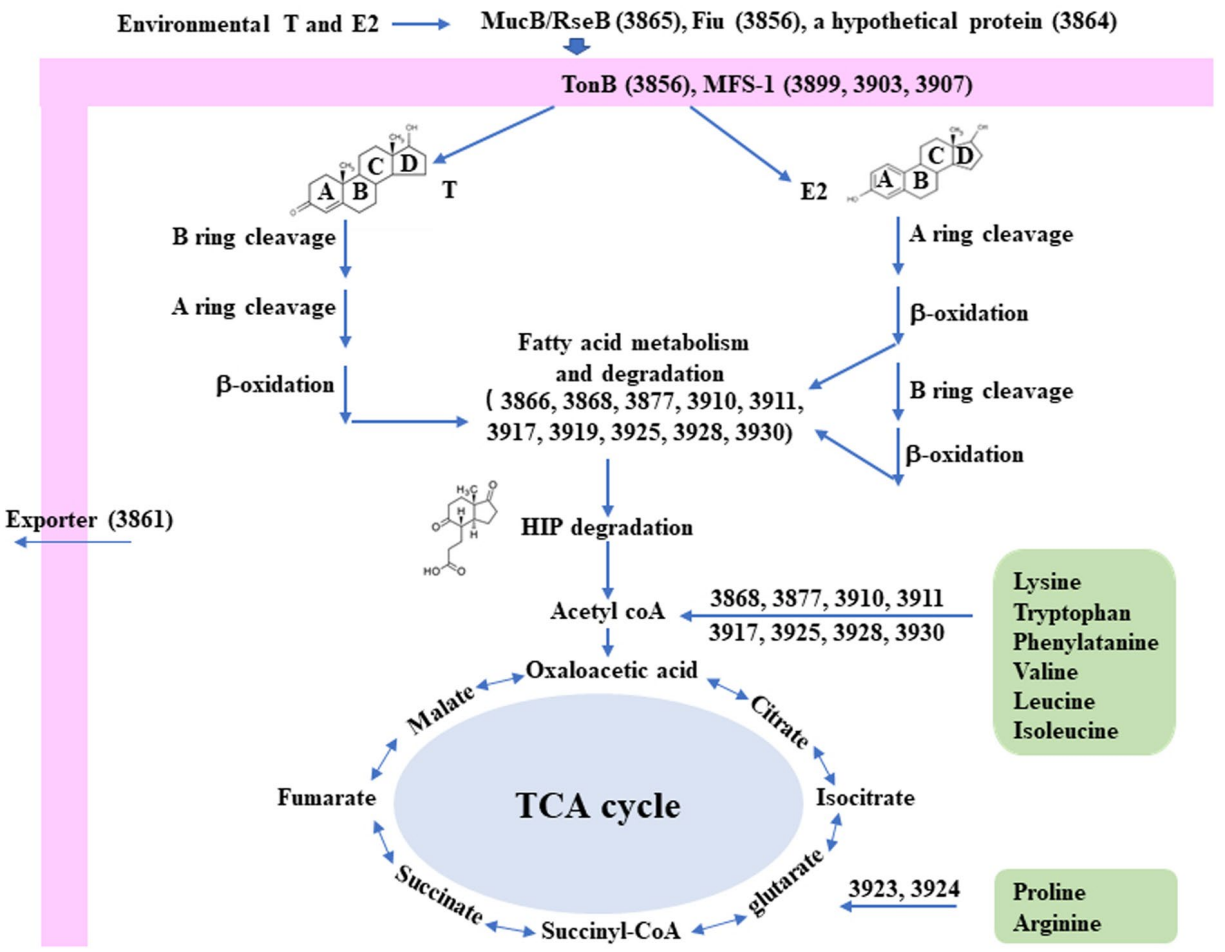
Diagram of the proposed metabolic mechanisms of the steroid degradation gene cluster that was significantly upregulated in *C. testosteroni* JLU460ET with testosterone (T) and 17 beta-estradiol (E2) induction. Number indicates gene ID. Information on key enzymes for steroid degradation is shown in the text and Table 4. The specific reaction process involved in this process has not been identified (Chen et al., 2017; Wu et al., 2019; Tian et al., 2020).

Interestingly, a significantly induced 100 kb steroid degradation gene cluster was found in the *C. testosteroni* JLU460ET genome, including 92 annotated genes (Table 4), 58 novel genes, and several sRNAs. The annotated genes were mainly involved in steroid degradation, aromatic compound degradation, fatty acid metabolism and degradation, and microbial metabolism in diverse environments. Three secretory proteins were found in this region in response to testosterone and 17 beta-estradiol in the environment: sigma E regulatory protein, MucB/RseB (3865), a kinase (3864), and outer membrane fiu catecholate siderophore receptor (3856). Gene 3,866 is a long-chain fatty-acid—CoA ligase for quorum sensing. Three copies of the major facilitator superfamily MFS_1 (3,899, 3,903, 3,907) membrane protein were found for the transport of testosterone and 17 beta-estradiol from the environment into *C. testosteroni* JLU460ET. Furthermore, three copies of steroid 3-ketoacyl-CoA thiolase genes (3,911, 3,917, and 3,925) work as a two-component system. In other reported articles, ABC transporters were responsible for steroid transport (Wu et al., 2019; Tian et al., 2020); in *C. testosteroni* JLU460ET, ABC transporters could be induced, but the induction level was very low, and they were not located in the steroid-inducible gene cluster. According to the analysis of upregulated genes located in the steroid-inducible gene cluster and other articles, the major facilitator superfamily (MFC-1) might be responsible for steroid transport in *C. testosteroni* JLU460ET (Pao et al., 1998; Nieng, 2015). The metabolic mechanism of the steroid degradation gene cluster that was significantly upregulated in *C. testosteroni* JLU460ET with testosterone and 17 beta-estradiol induction is presented in Figure 2. As shown in Figure 2, genes responsible for fatty acid metabolism and degradation, amino acid metabolism, and the TCA cycle were also inducible. These metabolic pathways are connected by acyl-CoA transferase, acyl-CoA dehydrogenase, acetyl-CoA C-acetyltransferase, acetyl-CoA transferase, acetyl-CoA dehydrogenase, enoyl-CoA hydratase/isomerase, thiolase, and 3-hydroxyacyl-CoA dehydrogenase. Four transcriptional regulators, such as LysR, LuxR, and XRE, were also found in the 100 kb steroid degradation gene cluster. They may control steroid degradation in *C. testosteroni* JLU460ET. The transcriptome results also showed that 59 novel genes located in this region were significantly induced by testosterone and 17 beta-estradiol. These inducible genes were first found in steroid degradation.

**Table 4:** Testosterone and 17 beta-estradiol degradation gene clusters found in *C. testosteroni* JLU460ET by transcriptome analysis.

Gene ID	Description	log2 fold change			
		E2-3 h induction	E2-13 h induction	T-3 h induction	T-13 h induction
GM003846	(*fadD*3) HIP---CoA ligase, long-chain-fatty-acid--CoA ligase	1.68	–	6.77	2.38
GM003847	(*kshB*) 3-ketosteroid-9-alpha-hydroxylase reductase	1.63	–	6.99	2.13
GM003848	(*tesI*) 3-oxo-5-alpha-steroid 4-dehydrogenase	2.25	–	7.04	1.70
GM003849	(*kstD*, *tesH*) 3-oxosteroid 1-dehydrogenase	2.28	–	7.08	1.67
GM003850	(*hsaA*) 3-hydroxy-9,10-secoandrosta-1,3,5(10)-triene-9,17-dione monooxygenase	2.02	–	7.63	2.52
GM003851	(*hsaB*) 3-hydroxy-9,10-secoandrosta-1,3,5(10)-triene-9,17-dione monooxygenase	2.44	–	7.77	2.72
GM003852	(*hsaD*) 4,5:9,10-diseco-3-hydroxy-5,9,17-trioxoandrosta-1(10),2-diene-4-oate hydrolase	2.27	–	8.48	3.45
GM003853	(*tesE*) 2-oxopent-4-enoate/cis-2-oxohex-4-enoate hydratase	2.52	–	8.23	3.40
GM003854	(*tesF*) acetaldehyde/propanal dehydrogenase	2.75	–	8.59	3.85
GM003855	(*tesG*) 4-hydroxy-2-oxovalerate/4-hydroxy-2-oxohexanoate aldolase	2.07	–	7.91	3.45
GM003856	TonB dependent receptor; Outer membrane receptor for monomeric catechols	–	–	2.57	–
GM003857	hypothetical protein	2.15	–	6.46	–
GM003858	Acyl dehydratase I Lipid transport and metabolism	1.62	–	6.97	3.07
GM003859	Acyl dehydratase I Lipid transport and metabolism	1.29	–	6.41	2.69
GM003860	Glycosyl hydrolase	3.01	0.92	8.41	2.34
GM003861	Exporter protein, integral to membrane	2.97	1.03	8.36	2.40
GM003862	3-phenylpropionate/cinnamic acid dioxygenase	2.54	–	7.81	1.60
GM003863	Dienelactone hydrolase	1.84	–	6.99	1.15
GM003864	Hypothetical protein	2.35	–	8.30	1.96
GM003865	Sigma E regulatory protein, MucB/RseB	2.27	–	7.91	–
GM003866	Long-chain-fatty-acid--CoA ligase	1.51	–	6.41	2.40
GM003867	Acyl-CoA dehydrogenase-like protein	1.43	–	6.20	2.05
GM003868	Acyl-CoA dehydrogenase-like protein	1.03	–	6.25	2.07
GM003869	Metal-dependent hydrolase	2.33	0.93	7.58	2.55
GM003870	Long-chain-acyl-CoA synthetase	2.88	1.18	8.38	3.17
GM003871	Acyl-CoA dehydrogenase FadE26	2.51	–	8.20	3.85
GM003872	Acetyl-CoA acetyltransferase I	2.66	1.20	8.19	3.87
GM003873	Acyl dehydratase I	3.15	–	8.40	3.69
GM003874	Acyl-CoA dehydrogenase	2.62	1.40	8.23	3.61
GM003875	Nucleic-acid-binding protein, contains Zn-ribbon domain	2.77	–	8.86	4.24
GM003876	3-beta hydroxysteroid dehydrogenase/isomerase family	2.57	1.48	7.83	3.34
GM003877	Enoyl-CoA hydratase	1.96	1.21	6.53	2.57
GM003878	Ribulose-phosphate 3-epimerase, carbon-sulfur lyase activity	–	–	1.36	–
GM003879	Short-chain alcohol dehydrogenase	–	–	0.45	–
GM003880	LysR family transcriptional regulator	–	–	–	–
GM003881	Acetyltransferase	–	–	–	–
GM003882	Hypothetical protein	–	–	4.40	0.99
GM003883	Regulator of nucleoside diphosphate kinase, Transcription elongation factor	0.74	–	3.34	–
GM003884	Delta-5-3-ketosteroid isomerase	1.30	–	6.67	–
GM003885	(*hsdA*)3alpha-hydroxysteroid 3-dehydrogenase	–	–	7.44	1.86
GM003886	(*fadH*)2,4-dienoyl-CoA reductase	2.40	1.21	5.77	1.80
GM003887	2,4-dienoyl-CoA reductase	1.75	1.22	5.81	2.22
GM003888	Hypothetical protein	–	–	–	–
GM003889	N-acetyltransferase	–	–	–	–
GM003890	Hypothetical protein	–	–	–	–
GM003891	3′-kinase	1.07	–	0.72	–
GM003892	Hypothetical protein	–	1.47	–	1.15
GM003893	Ketosteroid isomerase	–	–	–	–
GM003894	Metal-dependent hydrolase	1.08	1.16	1.00	0.92
GM003895	Protein-disulfide isomerase	1.60	1.68	0.94	1.43
GM003896	LysR family transcriptional regulator	1.12	0.94	1.40	0.85
GM003897	(*hdhA*)7-alpha-hydroxysteroid dehydrogenase	1.81	0.82	6.28	1.74
GM003898	Coniferyl aldehyde dehydrogenase	–	–	4.52	–
GM003899	Major facilitator superfamily MFS_1	1.55	1.37	4.69	2.38
GM003900	Short-chain dehydrogenase/reductase SDR	1.38	1.33	4.86	2.29
GM003901	(*kshA*) 3-ketosteroid 9alpha-monooxygenase subunit A	–	–	7.26	2.17
GM003902	Hypothetical protein	2.21	1.65	4.52	1.78
GM003903	Major facilitator superfamily MFS_1	2.34	1.24	5.82	1.50
GM003904	Amidohydrolase	1.83	1.33	4.23	1.11
GM003905	Hypothetical protein	–	–	1.94	–
GM003906	(*fadD*19) 3-oxocholest-4-en-26-oate---CoA ligase	–	–	4.37	–
GM003907	Major facilitator superfamily MFS_1	2.15	1.93	5.56	1.73
GM003908	Acyl-CoA dehydrogenase, oxidoreductase	1.69	1.64	5.34	1.65
GM003909	Choline dehydrogenase	1.96	2.06	5.63	1.99
GM003910	(*fadJ*) 3-hydroxyacyl-CoA dehydrogenase	1.60	1.34	5.69	1.62
GM003911	3-oxoacyl-[acyl-carrier-protein] synthase	2.19	1.92	6.24	2.03
GM003912	(*fabG*) 3-oxoacyl- [acyl-carrier protein] reductase	2.06	1.56	6.33	1.63
GM003913	Bile acid-inducible, Alpha-methylacyl-CoA racemase	1.66	1.36	5.48	1.66
GM003914	Two-component response regulator	–	–	–	–
GM003915	Hypothetical protein	–	–	–	–
GM003916	LuxR family transcriptional regulator	–	–	4.88	–
GM003917	3-ketoacyl-CoA thiolase	–	–	5.07	–
GM003918	Acyl dehydratase I Lipid transport and metabolism	–	–	5.57	–
GM003919	(*fabG*) 3-oxoacyl- [acyl-carrier protein] reductase	–	–	5.35	–
GM003920	Acyl-CoA dehydrogenase	1.44	1.16	5.95	2.15
GM003921	Acyl-CoA dehydrogenase	2.22	0.96	6.87	1.88
GM003922	Short-chain dehydrogenase	2.10	–	7.43	2.34
GM003923	Twin-arginine translocation pathway signal	2.06	–	6.83	1.81
GM003924	Twin-arginine translocation pathway signal	1.98	0.84	6.79	2.20
GM003925*	Steroid 3-ketoacyl-CoA thiolase	2.05	–	7.41	3.20
GM003926*	Acyl-CoA dehydrogenase	2.22	–	7.83	3.40
GM003927*	Acyl-CoA dehydrogenase	2.34	–	7.89	3.46
GM003928*	Enoyl-CoA hydratase	2.10	–	7.48	3.99
GM003929*	2-nitropropane dioxygenase	1.86	–	7.64	3.42
GM003930*	Enoyl-CoA hydratase	1.58	–	7.25	2.99
GM003931*	Acetate/3-ketoacid CoA transferase	2.76	–	8.65	3.65
GM003932*	Coenzyme A transferase	2.15	–	8.35	3.50
GM003933*	(*hsaC*) 3,4-dihydroxy-9,10-secoandrosta-1,3,5(10)-triene-9,17-dione 4,5-dioxygenase	2.44	–	8.25	3.83
GM003934	2,4-dienoyl-CoA reductase	–	–	5.88	2.63
GM003935	Hypothetical protein	–	–	6.75	2.61
GM003936	2,5-dichloro-2,5-cyclohexadiene-1,4-diol dehydrogenase	1.95	–	7.66	2.80
GM003937	(*fabG*) 3-oxoacyl- [acyl-carrier protein] reductase	–	–	6.90	2.39
GM003938	XRE family transcriptional regulator	–	–	–	–

“*” indicates genes selected for qRT-PCR validation.

### Gene expression validation by quantitative real-time PCR

**Figure 3:**
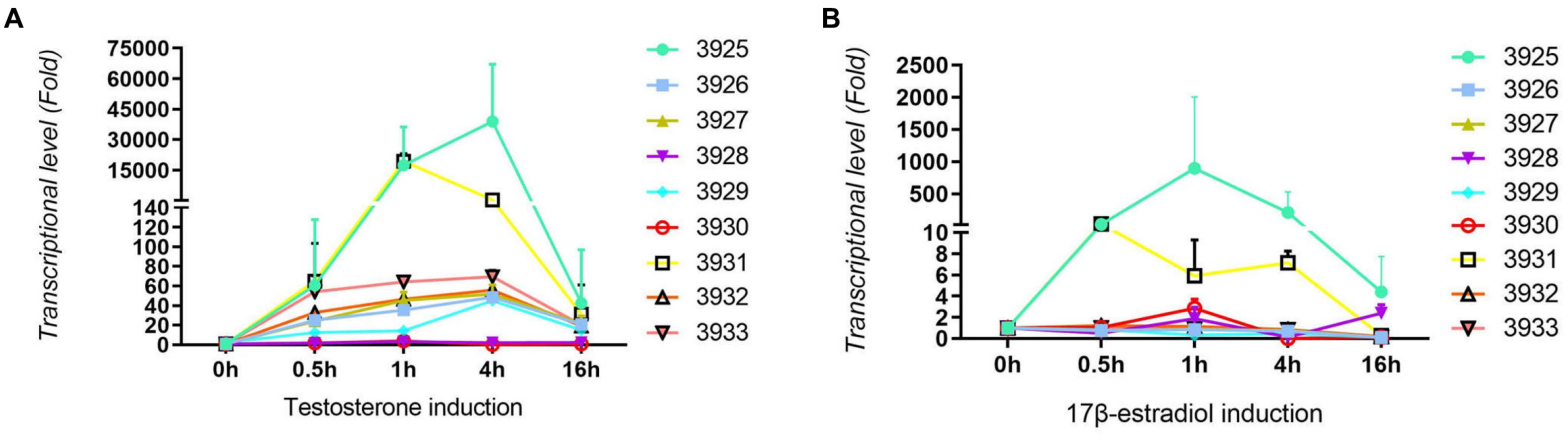
Results from qRT-PCR analysis of nine upregulated genes in *C. testosteroni* JLU460ET by transcriptome analysis. **(A)** *C. testosteroni* JLU460ET was incubated with testosterone; **(B)** *C. testosteroni* JLU460ET was incubated with 17 beta-estradiol.

For confirmation and validation of RNA-seq data by qRT–PCR, nine upregulated genes were selected. As shown in Figure 3A, all tested genes were significantly induced by testosterone. Most of the tested genes were induced approximately 60-fold with testosterone. Gene number 3925 is a steroid 3-ketoacyl-CoA thiolase, and gene number 3931 is a 3-ketoacid CoA transferase. Those two genes had the highest induction, i.e., more than 10,000-fold. Gene numbers 3,928 and 3,930 are enoyl-CoA hydratases, and they have relatively lower induction of less than 10-fold. As shown in Figure 3B, all tested genes were induced by 17 beta-estradiol, but the level of induction was approximately 1–4 times of the control without substrates, which was lower than testosterone induction. This might be the main reason why the degradation speed of testosterone is faster than that of 17 beta-estradiol. Genes 3,925 and 3,931 could also be significantly induced by 17 beta-estradiol. Genes 3,925 and 3,931 may be involved in the A-ring cleavage and degradation of testosterone and 17 beta-estradiol in the 3 position according to previous reports (Wu et al., 2019). The qRT-PCR results were consistent with the transcriptome sequencing results.

### Upregulated genes involved in testosterone and 17 beta-estradiol degradation by KEGG enrichment analysis

Both testosterone and 17 beta-estradiol are steroids. They share some common chemical structure features. The testosterone degradation pathway is very clear. While that of 17 beta-estradiol is not. Testosterone is much easier to biodegrade than 17 beta-estradiol. To identify common and unique genes involved in the testosterone and 17 beta-estradiol degradation pathways, all upregulated genes were analyzed.

**Figure 4:**
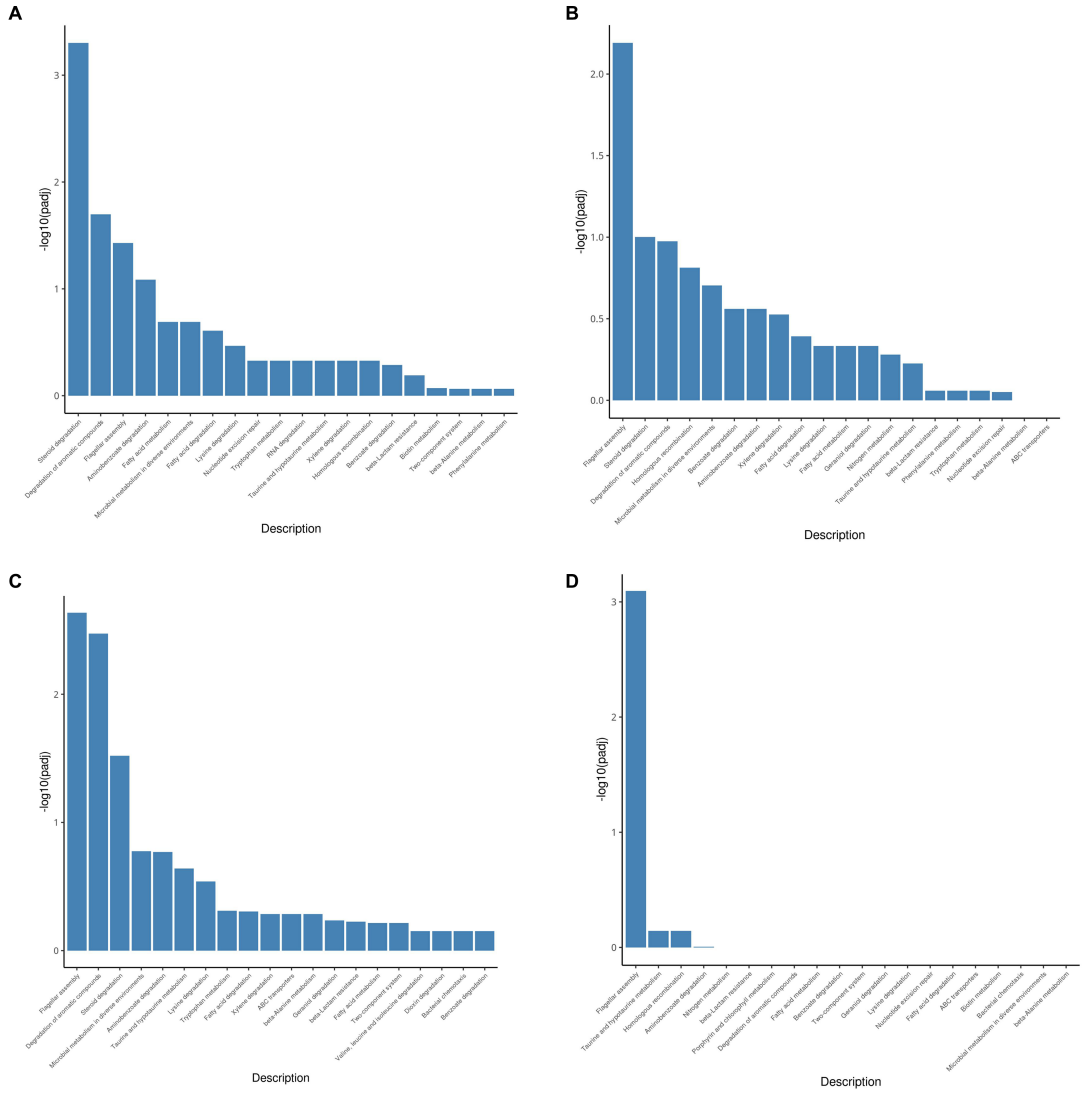
KEGG pathway enrichment analysis of testosterone and 17 beta-estradiol induction under different conditions. **(A)** 3-h induction with testosterone; **(B)** 13-h induction with testosterone; **(C)** 3-h induction with 17beta-estradiol; **(D)** 13-h induction with 17 beta-estradiol.

The KEGG pathway database is an extensive conventional source of molecular pathway maps. Mapping of the differentially expressed genes to the KEGG pathways may yield insights into the functional applicability of the genes analogous to high-throughput expression data. Thus, all genes upregulated by testosterone and 17 beta-estradiol were mapped with the KEGG database and categorized into many pathways (Figure 4). As shown in Figure 4, the upregulated genes were mainly involved in the steroid degradation pathway, aromatic compound degradation pathway, flagellar assembly, aminobenzoate degradation, fatty acid degradation, and microbial metabolism in diverse environments. Although testosterone, estrogens, and cholesterol are steroids, the estrogen degradation pathways remain unclear, so the steroid degradation pathway in the KEGG database is mainly biased toward cholesterol and testosterone degradation. Since the chemical structure of 17 beta-estradiol contains an aromatic ring, the E2 degradation pathway is similar to aromatic compound degradation. In this article, the upregulated genes involved in the steroid degradation pathway and aromatic compound degradation pathway were chosen for detailed analysis.

**Figure 5:**
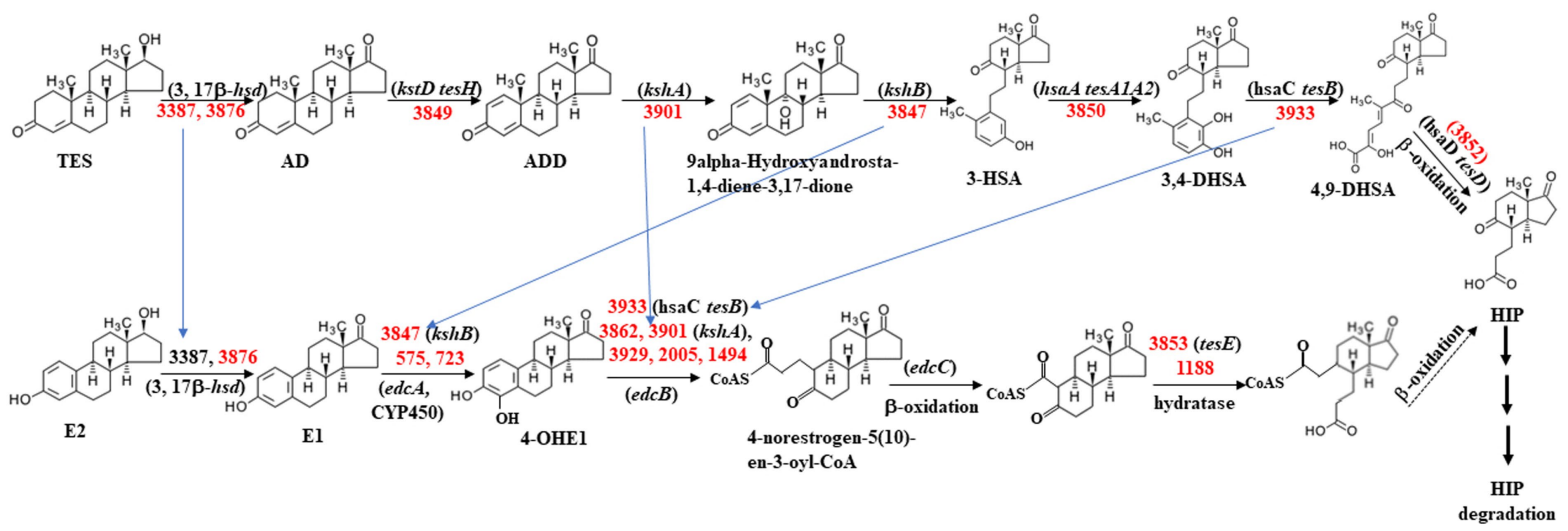
Proposed testosterone and 17 beta-estradiol degradation pathway in *C. testosteroni* JLU460ET according to map00984 in KEGG. Numbers indicate gene ID in *C. testosteroni* JLU460ET; red indicates significantly inducible. Compound names are shown or indicated with an abbreviation: (TES) testosterone; (AD) androstenedione; (ADD) Androsta-1,4-diene-3,17-dione; (3-HSA) 3-hydroxy-9,10-secoandrosta- 1,3,5(10)-triene-9,17-dione; (3,4-DHSA) 3,4-dihydroxy-9,10-secoandrosta-1,3,5(10)- triene-9,17-dione; (4,9-DHSA) 3-hydroxy-5,9,17-trioxo-4,5:9,10-disecoandrosta- 1(10),2-dien-4-oate; (E2) 17beta-estradiol; (E1) estrone; (4-OHE1) 4-hydroxyestrone; (HIP) 9,17-dioxo-1,2,3,4,10,19-hexanorandrostan-5-oic acid. Enzyme names are (3,17β-hsd) 3,17 β-hydroxysteroid dehydrogenase; (*kstD tesH*) 3-ketosteroid-delta1- dehydrogenase; (*kshA*) 3-ketosteroid 9alpha-monooxygenase; (*kshB*) 3-ketosteroid 9 alpha-hydroxylase; (*hsaA tesA1A2*) 3-hydroxy-9,10-secoandrosta-1,3,5(10)-triene- 9,17-dione monooxygenase; (*hsaC tesB*) 3,4-dihydroxy-9,10-secoandrosta-1,3,5(10)- triene-9,17-dione 4,5-dioxygenase; (*hsaD tesD*) 4,5:9,10-diseco-3-hydroxy-5,9,17- trioxoandrosta-1(10),2-diene-4-oate hydrolase; (*edcA*) E1 4-hydroxylase; (*edcB*) 4-OHE1 4,5-dioxygenase; (*edcC*) meta-cleavage product decarboxylase. The homologs *tesA ~ tesH* were from strain *C. testosteroni* CNB-2 and *C. testosteroni* TA441; *kstD*, *kshA*, *kshB*, and *hsaA ~ G* were from strain *M. tuberculosis* H37Rv and *Rhodococcus jostii* RHA1; *edcA ~ C* were from strain *N. tardaugens* NBRC 16725 (Chen et al., 2017; Ibero et al., 2019; Wu et al., 2019; Liu et al., 2020).

As shown in Table 5, approximately 10 genes involved in the steroid degradation pathway were upregulated. Approximately 25 genes involved in the aromatic compound degradation pathway were upregulated. As shown in Figure 5, most of the important steroid-degrading genes in the KEGG database (map00984) could be significantly induced by testosterone and 17 beta-estradiol in strain *C. testosteroni* JLU460ET. Importantly, all the upregulated key genes were located in the steroid-degrading gene cluster.

**Table 5:** Up-regulated gene numbers in KEGG pathways in *C. testosteroni* JLU460ET induction with testosterone or 17 beta-estradiol.

KEGG pathways	T induction-3 h	T induction-13 h	E2 induction-3 h	E2 induction-13 h
Steroid degradation	11	9	9	0
Degradation of aromatic compounds	25	24	26	19

“T” indicates testosterone; “E2” indicates 17 beta-estradiol.

Among all inducible genes, most were induced by both testosterone and 17 beta-estradiol. A total of 199 genes were solely induced by 17 beta-estradiol, including oxidoreductase, ABC transporter, fatty acid desaturase, ring-hydroxylating dioxygenase, membrane protein, methyl-accepting chemotaxis sensor, fatty-acid—CoA ligase, enoyl-CoA hydratase/isomerase, monooxygenase, metal-dependent hydrolase, and other proteins. A total of 246 genes were solely induced by testosterone, including long-chain fatty acid transport protein, 3-(or 17)-beta-hydroxysteroid dehydrogenase, 3-alpha-(or 20-beta)-hydroxysteroid dehydrogenase, and other proteins. 3-(or 17)-beta-hydroxysteroid dehydrogenase was reported to be a key enzyme for 17 beta-estradiol and testosterone degradation. In strain JLU460ET, two 3-(or 17)-beta-hydroxysteroid dehydrogenase genes were found in the genome (gene numbers 3,387 and 3,876). The transcriptome results of this study showed that gene 3,876 could be induced by both testosterone and 17 beta-estradiol, but gene 3,387 could only be induced by testosterone.

## Discussion

Endocrine disruptors are chemicals that interfere with the endocrine system and produce adverse effects in both humans and wildlife. Among all endocrine disruptors, 17 beta-estradiol (estrogen) and testosterone (androgen) are the most ubiquitously sexual hormones found as pollutants (Ibero et al., 2019). Microbial degradation is a crucial mechanism to eliminate them from contaminated systems. Previous studies reported that *Comamonas testosteroni* strains could degrade testosterone very fast, from which it gets the name (Liu et al., 2021a). Many scientists also tried to isolate highly effective 17beta-estradiol-degrading strains during the last 20 years. Unfortunately, many bacteria can only partially degrade/transform 17beta-estradiol, and only a few of them are able to completely metabolize 17 beta-estradiol and use it as the sole carbon and energy source. There are very few bacteria described so far that can catabolize both 17beta-estradiol and testosterone (Yu et al., 2013). Most importantly, the degradation rate of 17beta-estradiol is much slower than that of testosterone. For example, 272 mg/L testosterone could be degraded in 9 h by *C. testosteroni* JLU460ET, but 5 mg/L 17beta-estradiol needed approximately 5 days for complete degradation (Liu et al., 2021a). Testosterone and 17 beta-estradiol share similar chemical structure. Previous studies reported that some bacteria use the same gene products to degrade testosterone and 17 beta-estradiol (Chen et al., 2017; Ibero et al., 2019). Our group thought that maybe we can improve 17 beta-estradiol degradation rates by changing the degradation pathway of 17 beta-estradiol into the testosterone degradation pathway in strain *C. testosteroni* JLU460ET. In this article, whole-transcriptome analysis was finished to systematically characterize the global response of strain JLU460ET to 17 beta-estradiol and testosterone.

Whole-transcriptome analysis results showed that the number of upregulated genes was similar among the compared groups. Among all inducible genes, most were induced by both testosterone and 17beta-estradiol. Only 199 genes were solely induced by 17 beta-estradiol, and 246 genes were solely induced by testosterone. This result illustrated that *C. testosteroni* JLU460ET indeed used the same genes to degrade testosterone and 17 beta-estradiol. However, the upregulated log2-fold change value of 17 beta-estradiol was lower than those of testosterone; this may be the reason why strain *C. testosteroni* JLU460ET degrades testosterone faster than 17 beta-estradiol. Next, our group will try to find the key enzymes for 17 beta-estradiol degradation and improve the transcriptional level to increase the 17 beta-estradiol degradation rate.

The transcriptome analysis results also showed that a significantly induced 100 kb steroid degradation gene cluster was found in the *C. testosteroni* JLU460ET genome. Most annotated genes were mainly involved in steroid degradation, aromatic compound degradation, and fatty acid metabolism and degradation. We noticed that 58 novel genes were found in this steroid gene cluster by transcriptome analysis. More experiments will be conducted to explore the functions of those novel genes in steroid degradation. Several transposases were also found in the 100 kb steroid degradation gene cluster. This result illustrated that *C. testosteroni* JLU460ET probably obtained the steroid degradation gene cluster by horizontal gene transfer. In *C. testosteroni* JLU460ET, few significantly inducible genes are located outside the steroid degradation gene cluster. This is similar to another steroid degradation strain *N. tadaugens* ARI-1. In both strains *N. tadaugens* ARI-1 and *C. testosteroni* JLU460ET, only a few genes required for C-19degradation are located outside the steroid degradation cluster. It was thought that genes located outside the steroid degradation gene cluster might be involved in the aerobic degradation of other steroid compounds, so their location outside the cluster suggests that they played a more global role (Ibero et al., 2019).

3-(or 17)-beta-hydroxysteroid dehydrogenase genes are essential enzymes in the biosynthesis of all classes of mammalian steroids. They catalyze the interconversion of alcohol and carbonyl functions stereospecifically in defined positions using oxidized or reduced NAD(H) or NADP(H) as co-substrates (Straus, 2009; Ibero et al., 2019). It is also well known that 3-(or 17)-beta-hydroxysteroid dehydrogenase genes are involved in testosterone and 17 beta-estradiol degradation (Ibero et al., 2019). In *C. testosteroni* strains, 3-(or 17)-beta-hydroxysteroid dehydrogenase catalyzes the reversible reduction/dehydrogenation of the oxo/beta-hydroxy groups at positions C3 and C17 of steroids (Ibero et al., 2019). Two 3-(or 17)-beta-hydroxysteroid dehydrogenase genes were found in the genome of *C. testosteroni* JLU460ET. The transcriptome results of this study showed that one 3-(or 17)-beta-hydroxysteroid dehydrogenase gene (gene number 3876) could be induced by both testosterone and 17 beta-estradiol. This gene is located in the steroid degradation gene cluster. The other 3-(or 17)-beta-hydroxysteroid dehydrogenase gene (gene number 3387) could only be induced by testosterone. This gene is located outside the steroid degradation gene cluster. Although many studies have been conducted with the structure and functions of 3-(or 17)-beta-hydroxysteroid dehydrogenases (Benach et al., 1996; Oppermann et al., 1997; Benach et al., 2002; Straus, 2009), it is necessary to find out the reason that gene number 3387 could not be induced by 17 beta-estradiol in *C. testosteroni* JLU460ET in our future studies.

## Conclusion

In conclusion, (1) according to the results of transcriptome analysis in *C. testosteroni* JLU460ET, inducible genes involved in chemotaxis, the two-component system and transporters for both testosterone and 17 beta-estradiol are similar, and the degree of induction by those two substrates is also similar. (2) A 100 kb steroid degradation gene cluster was found in *C. testosteroni* JLU460ET by transcriptome analysis. Both testosterone and 17 beta-estradiol could induce the expression of genes located in the steroid-degrading gene cluster, but the induction by testosterone was much higher than that by 17 beta-estradiol. This could be the reason why *C. testosteroni* JLU460ET degrades testosterone faster than 17 beta-estradiol. In future, the difference in the binding and catalytic capacity for testosterone and 17 beta-estradiol of those steroid-degrading enzymes will be studied in detail. Engineering key enzymes may improve the degradation speed of 17 beta-estradiol in *C. testosteroni* JLU460ET. (3) Two 3-(or 17)-beta-hydroxysteroid dehydrogenase genes in *C. testosteroni* JLU460ET genome could be significantly induced by testosterone, but only one 3-(or 17)-beta-hydroxysteroid dehydrogenase gene could be induced by 17 beta-estradiol. (4) A methyl-accepting chemotaxis sensory transducer (GM002580) and several hypothetical proteins could be significantly induced only by 17 beta-estradiol not by testosterone. They might be specific for 17 beta-estradiol degradation. Their function in 17 beta-estradiol degradation in *C. testosteroni* JLU460ET will be researched in future studies.

## Data availability statement

The datasets presented in this study can be found in online repositories. The names of the repository/repositories and accession number(s) can be found in the article/Supplementary material.

## Author contributions

TZ is responsible for experimental design and paper writing, other authors are responsible for experimental operations. Most of the experiments were completed by ZW, MC, and NL. All authors contributed to the article and approved the submitted version.
