# Supplementary material for: Common and unique testosterone and 17 beta-estradiol degradation mechanisms in Comamonas testosteroni JLU460ET by transcriptome analysis
Source: Front Microbiol. 2023 Oct 26;14:1238855. doi: 10.3389/fmicb.2023.1238855 (PMC10637631; doi:10.3389/fmicb.2023.1238855)
Supplement: Supplementary file 1 [file Table_1.pdf]

Table S1 Summary table of total RNA extraction and test results

Sample names	Description	Concentration (ng/μL)	Volume (μL)	Total (μg)	RNA integrity
G1_3_1	without induction after 3 hours of incubation	49	32	1.568	9.1
G1_3_2		8	32	0.256	8.2
G1_3_3		17	32	0.544	9.6
G1_T_3_1	testosterone induction after 3 hours of incubation	20	32	0.64	10.0
G1_T_3_2		76	32	2.432	10.0
G1_T_3_3		31	32	0.992	9.1
G1_E2_3_1	17 beta-estradiol induction after 3 hours of incubation	492	32	15.744	10.0
G1_E2_3_2		183	32	5.856	9.9
G1_E2_3_3		522	32	16.704	10.0
G1_13_1	without induction after 13 hours of incubation	451	32	14.432	9.9
G1_13_2		364	32	11.648	9.9
G1_13_3		439	32	14.048	9.9
G1_T_13_1	testosterone induction after 13 hours of incubation	257	32	8.224	8.9
G1_T_13_2		233	32	7.456	9.2
G1_T_13_3		335	32	10.72	8.4
G1_E2_13_1	17 beta-estradiol induction after 13 hours of incubation	206	32	6.592	9.5
G1_E2_13_2		363	32	11.616	9.4
G1_E2_13_3		348	32	11.136	9.4
