# Supplementary material for: Common and unique testosterone and 17 beta-estradiol degradation mechanisms in Comamonas testosteroni JLU460ET by transcriptome analysis
Source: Front Microbiol. 2023 Oct 26;14:1238855. doi: 10.3389/fmicb.2023.1238855 (PMC10637631; doi:10.3389/fmicb.2023.1238855)
Supplement: Supplementary file 2 [file Table_2.pdf]

Table S2. Primers used for selected genes from *C. testosteroni* JLU460ET for qRT-PCR validation

Gene ID	primers name	Sequence (5'-3')
16S control	16S-RT-F	GTCTTCGGATGCTGACGAGT
	16S-RT-R	CTCTAGTAGCACAAGGCCCG
GM003925	3925-RT-F	TTTGGCTGCGTGGATACCAT
	3925-RT-R	TGGGCTGCAAAATGAATGGC
GM003926	3926-RT-F	GCAAGCAGTTCGACAAGGTC
	3926-RT-R	TAGAAGGCACGGTAGAGCAC
GM003927	3927-RT-F	GACCTATACCCCAGCGCAAA
	3927-RT-R	AACCCCTCTCGGGTGTCATA
GM003928	3928-RT-F	CATTGTGATCGCCGCAGAAG
	3928-RT-R	TTCTGCCGCACCAATCATCT
GM003929	3929-RT-F	TAGGTACGACCAATGCTGGC
	3929-RT-R	AAAGGCTGATCGTCGGTCTC
GM003930	3930-RT-F	GACATCCCCAAGCCCATGAT
	3930-RT-R	CTGAAAGAAGGCGTCATCGC
GM003931	3931-RT-F	CCAAGGTTTTTGTGTCGGGC
	3931-RT-R	AAGCGAATGTCGGTGGTCTC
GM003932	3932-RT-F	GCCAGAGTGGTGAGATCGAG
	3932-RT-R	GACGCGGGTGGGAATATAGG
GM003933	3933-RT-F	GTGATGGATCCGTCCGGAAA
	3933-RT-R	TAGTCGCCGGTGATAAAGCG
